# Molecular characterization of *S. japonicum* exosome-like vesicles reveals their regulatory roles in parasite-host interactions

**DOI:** 10.1038/srep25885

**Published:** 2016-05-13

**Authors:** Lihui Zhu, Juntao Liu, Jinwei Dao, Ke Lu, Hao Li, Huiming Gu, Jinming Liu, Xingang Feng, Guofeng Cheng

**Affiliations:** 1 Shanghai Veterinary Research Institute, Chinese Academy of Agricultural Sciences; Key Laboratory of Animal Parasitology, Ministry of Agriculture, 518 Ziyue Road, Shanghai, China

## Abstract

Secreted extracellular vesicles play an important role in pathogen-host interactions. Increased knowledge of schistosome extracellular vesicles could provide insights into schistosome-host interactions and enable the development of novel intervention strategies to inhibit parasitic processes and lessen disease transmission. Here, we describe biochemical characterization of *Schistosoma japonicum* exosome-like vesicles (*S. japonicum* EVs). A total of 403 proteins were identified in *S. japonicum* EVs, and bioinformatics analyses indicated that these proteins were mainly involved in binding, catalytic activity, and translation regulatory activity. Next, we characterized the population of small RNAs associated with *S. japonicum* EVs. Further studies demonstrated that mammalian cells could internalize *S. japonicum* EVs and transfer their cargo miRNAs to recipient cells. Additionally, we found that a specific miRNA, likely originating from a final host, ocu-miR-191–5p, is also associated with *S. japonicum* EVs. Overall, our findings demonstrate that *S. japonicum* EVs could be implicated in the pathogenesis of schistosomiasis via a mechanism involving the transfer of their cargo miRNAs to hosts. Our findings provide novel insights into the mechanisms of schistosome-host interactions.

Exosomes are membrane-bound vesicles that are constantly secreted by various types of mammalian cells in normal and disease states^1^,^2^. These vesicles are synthesized through reverse budding of the late endosomal membrane, resulting in the formation of multivesicular bodies^3^,^4^. Upon fusion of multivesicular bodies with the plasma membrane, exosomes are released into the extracellular environment and can be taken up by neighboring cells, undergo degradation, or enter bodily fluids and traffic to distal sites within the body^1^,^2^. Notably, exosomes comprise functional proteins, lipids, miRNAs, and mRNAs, which can transduce signals in target cells^2^,^5^. Exosomes play an important role in cell-cell communication and have been implicated in the regulation of many physiological processes, including cell development, immune regulation, angiogenesis, and cell migration^5^,^6^. In addition, circulating exosomes and their miRNA cargo are potential biomarkers of certain diseases^7^.

The genus *Schistosoma* is the causative agent of schistosomiasis, a neglected tropical infection that is endemic to developing countries and leads to approximately 300,000 deaths annually^8^. Most at-risk and infected individuals are concentrated in sub-Saharan Africa^9^. In China, schistosomiasis japonica is a serious public health problem, although tremendous efforts have been made to control its transmission over the last few decades^10^. Studies on schistosome biology have led to significant improvement in understanding the relationship between the schistosome and host^11^,^12^. However, a detailed elucidation of the mechanisms of parasitism is still required for identifying new drug targets, vaccine candidates, and biomarkers to control and diagnose schistosomiasis.

Exosomes are involved in cell-cell communication in many eukaryotes and prokaryotes^2^,^13^. Several recent studies have shown that protozoa can also release vesicles into the extracellular space, which can either dampen or elicit inflammatory responses in the host^14^,^15^,^16^. Additionally, studies on *Leishmania, Heligmosomoides polygyrus*, *Cryptococcus*, and *Trypanosoma* have demonstrated that vesicles can carry and deliver virulence factors (proteins and small RNAs) to the host, eliciting biological effects^14^,^17^,^18^. Characterization of extracellular vesicles secreted by *S. mansoni* schistosomula^19^ and adult worms^20^ suggested that extracellular vesicles and their cargo could play an important role in host-pathogen interactions. Wang *et al*.^21^ recently identified exosome-like vesicles (EVs) in *S. japonicum* but the EV components were not characterized^21^. Detailed characterization of schistosome vesicles could provide a greater understanding of their functions in pathogen-host interaction. Increased knowledge of these vesicles could also lead to the development of novel strategies to control schistosomiasis and the identification of novel biomarkers for schistosomiasis diagnosis.

We conducted the first attempt to conduct proteomic characterization of *S. japonicum* EVs, resulting in the identification of 403 proteins. We also identified a population of small RNAs associated with *S. japonicum* EVs. Functional studies demonstrated that secreted EVs are internalized by mammalian cells and can transfer their cargo miRNAs to recipient cells. These results demonstrate the regulatory potential of *S. japonicum* EVs in parasite-host interactions.

## Results

### Exosome isolation and proteomic characterization of *S. japonicum* EVs

**Figure 1:**
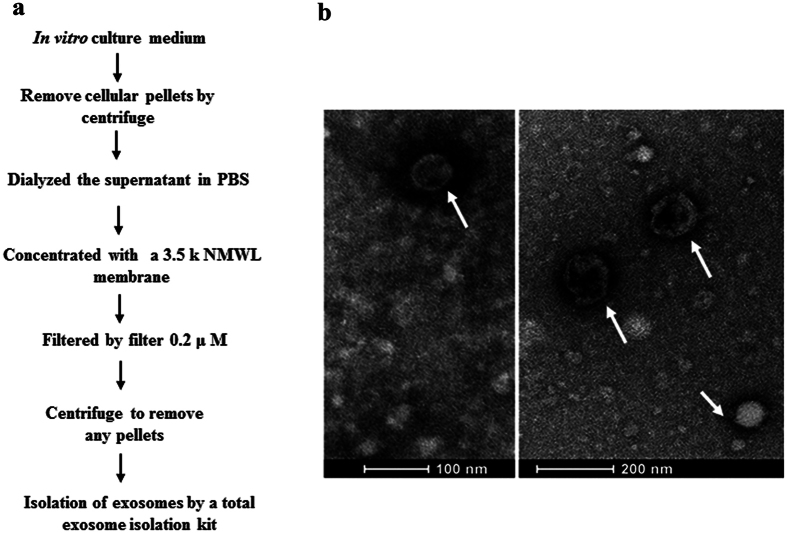
(**a**) Flow chart of *S. japonicum* EVs isolation from cultured medium. (**b**) Morphological characterization of *S. japonicum* EVs. Arrows indicate isolated *S. japonicum* EVs stained using uranyl acetate.

We developed a protocol for exosome isolation using dialysis, ultrafiltration, and ultracentrifugation combined with a commercial kit (Fig. 1a). Isolated EVs were subjected to morphological analyses using transmission electron microscope. The EVs showed round-shaped vesicles of approximately 50–100 nm in diameter (Fig. 1b). The protein components of *S. japonicum* EVs were determined by shotgun LC-MS/MS analyses. All MS/MS spectra were searched against a database assembled with *S. japonicum* sequences, and proteins identified with two or more peptides were further analyzed. In total, there were 2,024 non-redundant peptides detected, which corresponded to 403 proteins (Supplementary Dataset 1).

**Figure 2:**
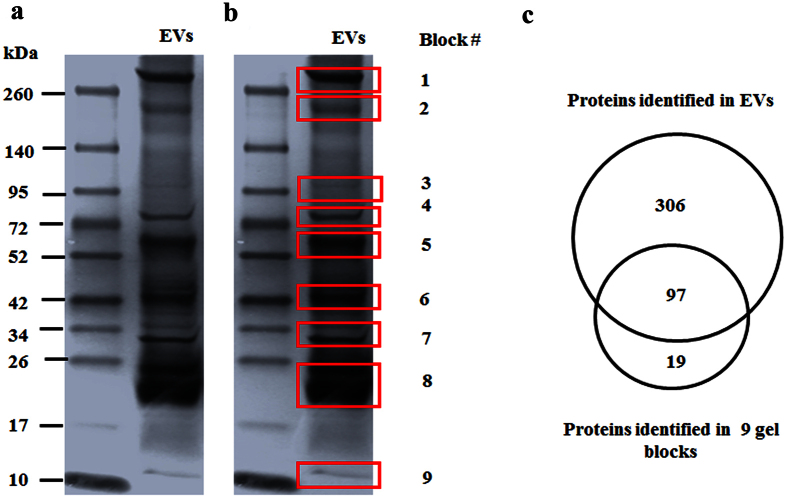
Proteomic analyses of *S. japonicum* EVs. (**a**) SDS-PAGE analyses of purified *S. japonicum* EVs using silver-staining. (**b**) Diagram of gel blocks excised for MS analyses. (**c**) Venn diagrams of identified proteins in *S. japonicum* EVs.

To further corroborate the results, we cut out nine main bands of gel blocks as displayed in the SDS-PAGE gel stained with silver (Fig. 2a). These gel blocks were subjected to in-gel digestion, and then the extracted peptides were analyzed by shotgun LC-MS/MS. MS results indicated that there were 116 proteins identified from the total of 9 gel blocks (Supplementary Dataset 2). Among them, 97 out of 116 proteins identified in the gel blocks were also detected in *S. japonicum* EVs by direct shotgun LC-MS/MS analyses (Fig. 2b). Further analyses indicated that 19.35% of the identified proteins (78 proteins) are homologous to the exosomal proteins in other parasites (Supplementary Dataset 1). In addition, we observed that 23 proteins in *S. japonicum* EVs showed high identities (>60%) with exosomal proteins from mammalian cells and other parasites (Table 1). We also compared the protein dataset with identified proteins from *S. japonicum* excretory/secretory products^22^ and *S. mansoni* extracellular vesicles^19^. We found that 12.66% (51 proteins) of the identified proteins were detected in *S. japonicum* excretory/secretory products. In addition, 31 proteins (7.69%) showed high identities (>69%) with those identified in *S. mansoni* exosomes (Supplementary Dataset 1).

**Figure 3:**
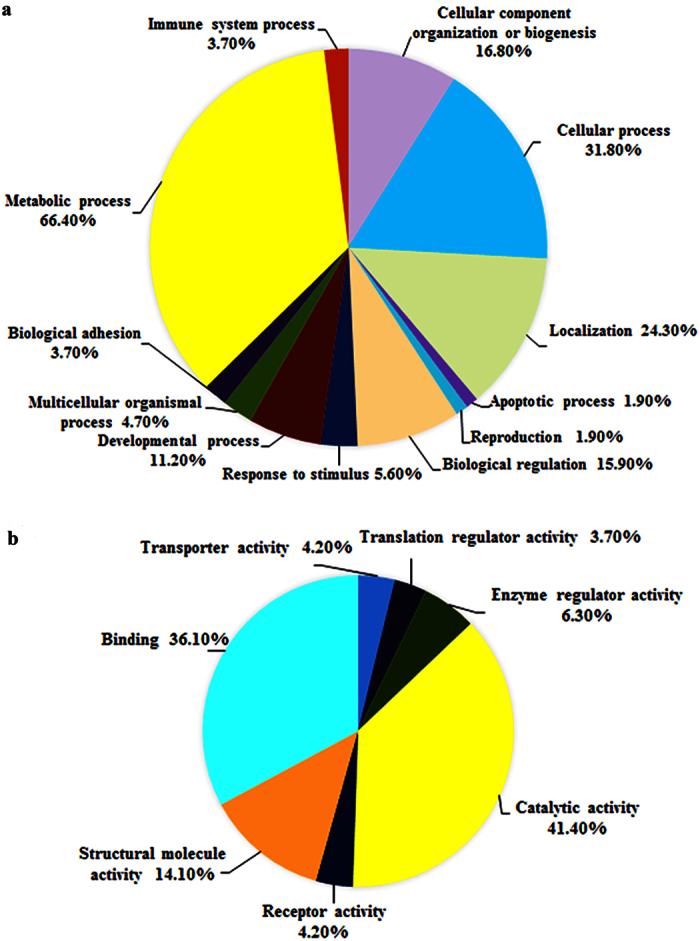
Gene ontology (GO) analysis of identified proteins in *S. japonicum* EVs. Pie diagram represents mapped protein count versus total identified proteins as a function of all available GO terms for biological process (**a**), or molecular function (**b**).

Next, we predicted the functions of *S. japonicum* EV proteins by bioinformatics analyses. We found that 191 proteins could be assigned to 216 gene ontology (GO) terms for biological processes. The top five GO categories were metabolic process, cellular process, localization, biological regulation, and developmental process (Fig. 3a). The prominent GO terms for molecular function were binding and catalytic activity, in accordance with the finding that many proteins were involved in metabolic processes (Fig. 3a,b). Bioinformatics analyses also indicated that 27 proteins (14.10% of the 191 proteins) are involved in structural molecule activity including elongation factor 1-gamma, twinfilin-1, tubulin gamma-1 chain, actin-related protein 2/3 complex subunit 4, *et al*. (Supplementary Table S1). In addition, 59 proteins (14.64%) were assigned to Kyoto Encyclopedia of Genes and Genomes analysis, and these assigned proteins are mainly involved in the integrin signaling pathway, CCKR signaling map, ubiquitin proteasome pathway, cytoskeletal regulation by Rho GTPase, disease-related pathways (Huntington disease and Parkinson disease), and inflammation mediated by chemokine and cytokine signaling pathways (Supplementary Table S2).

Moreover, we compared the identified proteins with the protein dataset from schistosome membrane and tegument. A total of 50 proteins (12.41%) were detected in the schistosome tegument (Supplementary Table S3)^20^,^23^ including 9 proteins putatively involved in membrane trafficking such as coatomer subunit beta, coatomer subunit gamma, coatomer subunit delta, sorting nexin-6, sorting nexin-18, protein SEC13 homolog, AP-1 complex subunit beta-1, syntaxin-binding protein 1, and syntaxin-binding protein 1. Furthermore, we compared our datasets with the data in ExoCarta database (http://exocarta.org/#). We found that 132 out of 403 proteins show homolog with the proteins deposited in ExoCarta including some exosomal markers such as CD63, heat-shock proteins (HSPs 70 and 90), actin, T-complex protein 1, tubulin, glyceraldehyde 3-phosphate dehydrogenase, Annexin, elongation factor, and Rab (Supplementary Dataset 1).

### Identification of small RNAs associated with *S. japonicum* EVs

**Figure 4:**
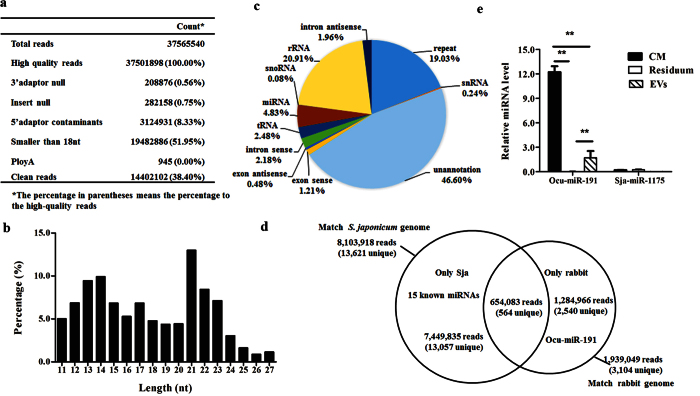
Identification of small RNAs associated with *S. japonicum* EVs. (**a**) Summary of the output of the Solexia data. (**b**) The length distribution of small RNAs. (**c**) Classification of the small RNAs that are annotated in the *S. japonicum* genome. (**d**) Venn diagrams showing small RNAs specifically matched to the *S. japonicum* and rabbit genomes. (**e**) qRT-PCR validation of the abundance of ocu-miR-191-5p in the RNA isolated from *S. japonicum* EVs. Results represent mean ± SEM from triplicate independent experiments. *P ≤ 0.05 and **P ≤ 0.01 (Tukey’s test, one-way ANOVA). CM: culture medium; Residuum: supernatant medium from the final step of EVs extraction.

Small RNAs incorporated into exosomes play a regulatory role in exosome-mediated cell-cell communication. Therefore, we identified the small RNA population associated with *S. japonicum* EVs using Solexa deep sequencing. The length distribution of *S. japonicum* EV-associated small RNAs ranged from 13 nt to 27 nt (Fig. 4a,b) and rRNAs, repeat associated small RNAs, and miRNAs were the dominant class of small RNAs (Fig. 4c). We found that 15 known *S. japonicum* miRNAs (cut-off reads >100) were present in *S. japonicum* EVs libraries (Table 2 and Supplementary Dataset 3), including two miRNAs (Bantam and miR-10) identified in the plasma of *S. japonicum* infected hosts in our previous study^24^. We also predicted 19 novel miRNAs using Mireap (Supplementary Dataset 4). In addition, there are 4 miRNAs associated with *S. japonicum* EVs (cut-off reads >100) homologous to miRNAs in ExoCarta database (Supplementary Table S4). Interestingly, we found that there were 1,284,966 reads (3.4%, 2,540 unique small RNAs) specifically mapped to the rabbit genome, including one miRNA, ocu-miR-191-5p, with a relatively high number of reads (4,548 reads) (Fig. 4d and Table 2). Next, we used stem-loop based qRT–PCR to further verify the present of ocu-miR-191-5p in the RNAs isolated from *S. japonicum* EVs (Fig. 4e). These findings suggested that *S. japonicum* EVs could carry both host and schistosome factors that could be involved in the pathogenesis of schistosomiasis and/or the regulation of schistosome development.

### Analyses of antibody responses to *S. japonicum* EVs

**Figure 5:**
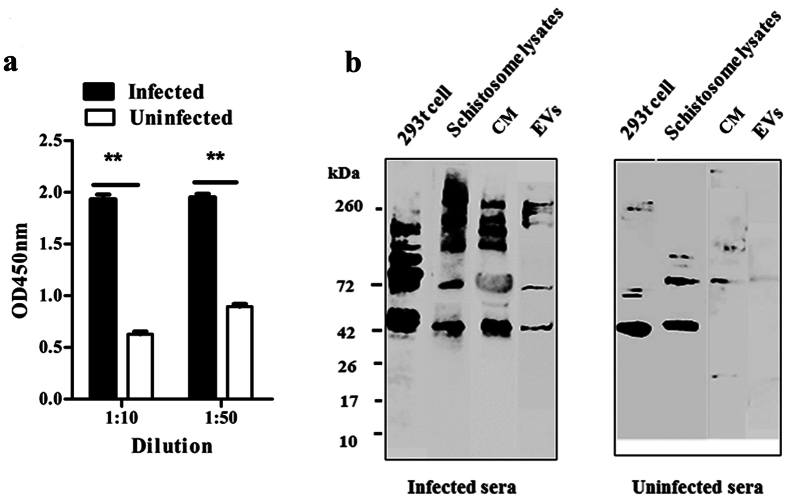
Immunological analyses of *S. japonicum* EVs recognized by sera collected from rabbits infected with *S. japonicum* cercariae. (**a**) ELISA analyses of antibody responses to *S. japonicum* EVs. Results represent mean ± SEM from triplicate experiments. **P ≤ 0.01 (Student’s t-test). (**b**) Western blot analyses of antibody responses to *S. japonicum* EVs. Each sample (30 μg) was separated by SDS-PAGE, electrotransferred, and probed with pooled sera of *S. japonicum* infected rabbits and uninfected rabbits. CM: culture medium.

To evaluate immune responses against *S. japonicum* EVs in a final host, we used enzyme-linked immunosorbent assay (ELISA) and Western blot to examine immunological recognition of *S. japonicum* EVs. As shown in Fig. 5, sera from infected rabbits indicated significant detection of *S. japonicum* EV proteins as compared with that of controls (Fig. 5a). Western blot analyses showed that sera from infected rabbits recognized multiple EV bands, whereas only a few bands were observed when applying uninfected serum (Fig. 5b). Reactive bands in the EVs were also different from those present in the 293 T cell preparations, which were detected with both infected serum pools and uninfected pools suggesting that infected sera could specifically recognize some proteins from *S. japonicum* EVs. These results indicate that schistosomes secrete EVs into the circulation of the final host and that secreted *S. japonicum* EVs and their protein components could be potential biomarkers for schistosomiasis diagnosis.

### Internalization of *S. japonicum* EVs in mammalian cells

**Figure 6:**
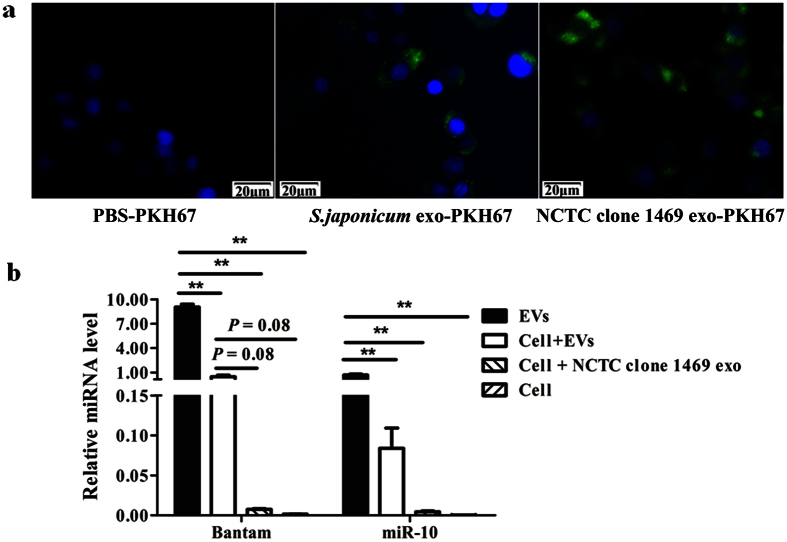
Uptake of *S. japonicum* EVs by murine liver cells. (**a**) Uptake of *S. japonicum* EVs by normal mouse liver cells detected using fluorescence microscopy. Approximately 10 μg of PKH67-labelled *S. japonicum* EVs, NCTC clone 1469 exosomes, and a PKH67-PBS control were added to the cells. Uptake of the labeled exosomes by NCTC clone 1469 cells was detected using fluorescence microscopy. Nuclei were stained with DAPI (blue). (**b**) qRT-PCR analysis of *S. japonicum* EV associated miRNAs in treated cells. Results represent mean ± SEM from triplicate experiments. *P ≤ 0.05 and **P ≤ 0.01 (Tukey’s test, one-way ANOVA).

Exosome cargo, especially miRNAs has been shown to play a regulatory role in host-pathogen interactions. To reveal potential roles of *S. japonicum* EVs in host-pathogen interactions, we labeled *S. japonicum* EVs with the lipid dye PKH67 and incubated the labeled exosomes with mouse liver cells (NCTC clone 1469 cells) *in vitro*. *S. japonicum* EVs were internalized by the NCTC clone 1469 cells (Fig. 6a). Further analyses demonstrated that *S. japonicum* EV associated miRNAs (Bantam and miR-10) were significantly detected in the RNA isolated form cells treated with labeled *S. japonicum* EVs (Fig. 6b), indicating that the miRNA cargo of *S. japonicum* EVs can transfer to recipient cells.

### qRT-PCR analysis of the expression of potential target mRNAs of *S. japonicum* EVs associated miRNA in mice

**Figure 7:**
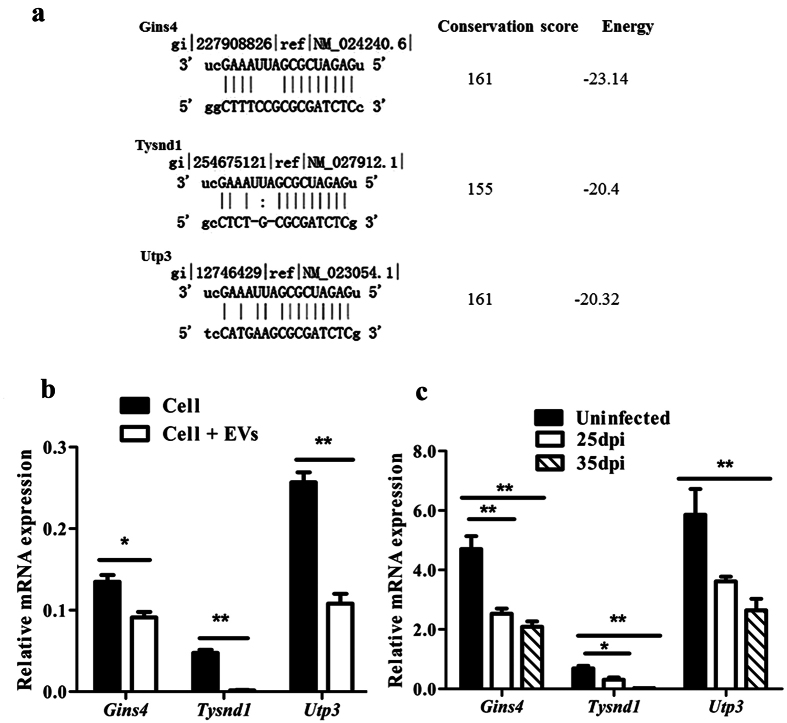
qRT-PCR analyses of the expression of mouse cDNAs that are potentially regulated by *S. japonicum* EV associated Bantam miRNA. (**a**) Bioinformatics analysis of the target genes of Bantam miRNA in mice. (**b**) qRT-PCR analyses of the expression of three potential target cDNAs of schistosome bantam miRNA in murine liver cells treated with *S. japonicum* EVs. Results represent mean ± SEM from triplicate experiments, *P ≤ 0.05 and **P ≤ 0.01(Student’s t-test). (**c**) qRT-PCR analyses of the expression of three potential target cDNAs of schistosome Bantam miRNA in liver of mice infected with *S. japonicum* cercariae (25 dpi: at 25 d post infection; 35 dpi: at 35 d post infection). *Gins4*: GINS complex subunit 4 (Sld5 homolog); *Tysnd1*: trypsin domain containing 1 (*Tysnd1*); *Utp3*: small subunit (SSU) processome component, homolog (*S. cerevisiae*). *P ≤ 0.05 and **P ≤ 0.01 (Tukey’s test, one-way ANOVA).

To determine potential regulatory roles of the cargo miRNA of *S. japonicum* EVs in host cells, we performed bioinformatics analyses to predict the target mRNAs for a specific *S. japonicum* associated miRNA, Bantam miRNA. Three murine genes (*Gins4*, *Tysnd1*, and *Utp3*) were shown to be potential targets of Bantam miRNA in mice (Fig. 7a). We analyzed the expressions of these three mRNAs in murine liver cells (NCTC clone 1469 cells) treated with *S. japonicum* EVs. All three genes were significantly (*P* < 0.05) down-regulated (Fig. 7b). To further corroborate these results, we determined the expressions of these three genes in the livers of mice infected with *S. japonicum* cercariae at 25 and 35 d post infection (dpi). We observed significantly (*P* < 0.05) down-regulated expression of these genes in animal level (Fig. 7c). Overall, these results indicated that uptake of *S. japonicum* secreted EVs could potentially regulate mouse genes via *S. japonicum* originating miRNAs.

## Discussion

Exosomes have been shown to act as signals for the membranes of target cells, participate in pathogen dissemination, and exert effects in host cells, including those of the host immune system^15^,^25^,^26^. However, knowledge of *S. japonicum* EVs is still limited, although a recent study indicated the existence of EVs derived from *S. japonicum* adult worms^21^. The proteins and miRNA cargo in *S. japonicum* EVs remain uncharacterized. It also remains unknown whether *S. japonicum* EVs can be internalized by mammalian cells and transfer miRNAs to recipient cells. In the present study, we developed a protocol to isolate *S. japonicum* EVs and then characterized the protein components of *S. japonicum* EVs and their associated small RNA population. We demonstrated that *S. japonicum* EVs can be internalized by mammalian cells and transfer their associated miRNAs to recipient cells.

Our proteomic data indicated that *S. japonicum* EVs included characteristic markers of exosomes, such as heat-shock proteins (HSPs 70 and 90), actin, elongation factor, and Rab proteins. Most proteins identified in the secretome of *Leishmania*, helminths, and other parasites were also observed in *S. japonicum* EVs, suggesting that exosome-like vesicles could play similar roles in protozoa and helminths^19^,^26^,^27^,^28^. We found that some proteins involved in vesicle biogenesis, such as heat-shock proteins (HSPs 70 and 90) and members of the Rab GTPase family (Rab10, Rab11, and Rab8), were also detected in *S. japonicum* EVs. Rab proteins are essential regulators of intracellular vesicle transport between different subcellular compartments via processes including vesicle budding, mobility along the cytoskeleton, and membrane tethering and fusion^29^. These results highlight the hypothesis that extracellular vesicle formation is a highly conserved mechanism in eukaryotes^30^ and could constitute an important mechanism for protein export in schistosomes.

Functional categorization of the identified EV proteins revealed a high proportion of nucleic acid-binding proteins, consistent with proteins identified in *Trypanosoma cruzi* and *Leishmania* secretomes^26^,^27^. An important inclusion to this category of proteins was RNA-binding proteins, including eukaryotic translation factors that have been assigned functions in both initiation and elongation and are known to play essential roles in gene expression regulation, modulating mRNA turnover, nucleocytoplasmic transport, and transcription^31^. Among these, eukaryotic translation elongation factors 1 alpha (eEF1A), are not only translation factors but also pleiotropic proteins highly expressed in tumors^32^. Additionally, data suggest that eEF1A proteins could not only activate the phospholipid and Akt signaling pathways that favor cell survival, but also block apoptosis and promote viral replication^33^. Thus, the release of such proteins suggests a regulatory role for *S. japonicum* EVs in parasite-host interaction and *S. japonicum* evasion from the host immune system.

Bioinformatics analyses indicated that exosome proteins could be involved in many pathways, including several already described in other parasites, such as the ubiquitin-proteasome system^34^. Among eukaryotes, the turnover of intracellular proteins is primarily mediated by the ubiquitin-proteasome system^35^, which is highly conserved from yeast to humans. Proteasomes are important for the survival and development of schistosomes^36^,^37^. In this study, the enrichment of proteasomes in *S. japonicum* EVs suggested that the ubiquitin-proteasome system could play an important regulatory role during schistosome infection. Proteasomal targeting could be a candidate strategy for anti-schistosome therapy due to the indispensable role of proteasomes in parasite invasion.

We also identified several schistosome surface antigens in the *S. japonicum* EVs, such as a 22.6 kDa tegumental antigen (AAC67308), tegument antigen (I(H)A)(CAX71406), and major egg antigen (p40, CAX78232), which have been implicated in schistosome evasion of host immune responses^38^,^39^. Indeed, the parasite tegument covers the surface of the worms, constitutes a major interface between the parasite and its host, and plays a critical role in host-parasite interactions^40^,^41^. Parasite molecules expressed at the tegument surface are potential targets for immune or drug intervention^41^,^42^. Similarly, tegumental proteins of schistosomes are also considered potential targets for schistosomiasis control^23^,^41^,^42^. We observed a high proportion of membrane- and tegument-associated proteins in *S. japonicum* EVs. In addition, significant immune reactivity against *S. japonicum* EVs was observed when applying sera from *S. japonicum*-infected rabbits. Our results provide a basis for the identification of potential target molecules for the development of vaccines and schistosomiasis biomarkers.

One of the mechanisms of exosomes mediating cellular communication is through miRNAs that can be transported by EVs and exert regulatory functions in recipient cells^43^,^44^. We demonstrated that mammalian cells could internalize *S. japonicum* EVs and their associated miRNAs. These results suggest that *S. japonicum* EVs potentially function as signal messengers that regulate host gene expression, which could facilitate schistosome parasitism. In *Drosophila*, Bantam miRNA has been shown to target a tumor-suppress pathway, leading to cellular growth and the suppression of cellular death^45^. In the present study, we found an enriched Bantam in *S. japonicum* EVs, which can be transferred to liver cells via *S. japonicum* EVs. Consequently, we hypothesized that schistosome-specific miRNAs, such as Bantam, may be involved in the hepatic pathogenesis of schistosomiasis. In support of this, we determined the mRNA expression of three potential target genes (*Gins4*, *Tysnd1*, and *Utp3*) of schistosome Bantam miRNA in mice. Both *in vivo* animal studies and *in vitro* cell culture study clearly indicated a common down-regulation of mRNA expressions in the livers of *S. japonicum* infected mice (28 dpi and 35 dpi) and in liver cells treated with *S. japonicum* EVs. In addition, we observed that a considerable number of small RNAs isolated from *S. japonicum* EVs (1,284,966 reads, 3.43%) specifically mapped to the rabbit genome, including a known miRNA (ocu-miR-191-5p) with a relatively high number of reads (4,548 reads); further qRT-PCR analysis indicated that ocu-miR-191-5p was also enriched in the RNAs isolated from *S. japonicum* EVs. These results suggest that *S. japonicum* EVs could be important regulators of host-pathogen interactions.

## Conclusion

We characterized the biochemical components of *S. japonicum* EVs using a proteomics approach and determined their associated small RNA populations using deep sequencing. We demonstrated that *S. japonicum* EVs, which contain parasite antigens, miRNAs, and potential virulence factors, can be internalized by host cells and transfer cargo miRNAs to recipient cells. These findings indicate that *S. japonicum* EVs may play an important regulatory role in parasite-host interactions and could be involved in the pathogenesis of schistosomiasis. Our findings provide novel insights into host-pathogen interactions that could lead to the development of novel therapies and diagnostic biomarkers for schistosomiasis.

## Methods

### Ethics statement

This study was performed in strict accordance with the recommendations of the Guide for Care and Use of Laboratory Animals of the National Institutes of Health. The protocol was approved by the Ethics and Animal Welfare Committee of the Shanghai Veterinary Research Institute, Chinese Academy of Agricultural Sciences.

### Parasite cultures and cultured medium collection

New Zealand rabbits were percutaneously infected with approximately 1,500 *S. japonicum* cercariae (Anhui isolate, China). Schistosomes at the liver stage were collected from rabbits infected with *S. japonicum* at 28 dpi. Parasites were thoroughly and gently washed three times with 50 mL PBS (pH 7.4) and then maintained in preheated RPMI-1640 culture medium (HyClone, Logan, UT) containing 100 U of penicillin and 100 mg/mL of streptomycin (Sigma, St. Louis, MO, USA) at 37 °C under 5% CO_2_ at a density of ~5 worm pairs /mL for 2 h. Following 2 h incubation, worms were microscopically examined to ensure their teguments were intact. Then, parasites and pellets were removed by centrifugation at 2,000 × *g* and 14,000 × *g* for 30 min each at 4 °C, respectively. The culture medium was collected, dialyzed in PBS for 24 h at 4 °C, and concentrated by centrifugal ultrafiltration through a 3 K NMWL membrane (Merck Millipore, Darmstadt, Germany). The supernatant was then filtered using a 0.22 μm syringe filter (Merck Millipore) and transferred to a 15 mL polyallomer tube for further exosome isolation using a total exosome isolation kit as described below. In addition, proteins from the culture medium without exosome isolation were directly precipitated by adding five volumes of cold acetone; the mixture was stored at −20 °C overnight.

### Isolation of exosome-like vesicles

A total exosome isolation kit (Life Technologies, Carlsbad, CA, USA) was used for exosome isolation according to the manufacturer’s instructions with minor modifications. All procedures were performed at 4 °C. In brief, the conditioned medium treated as described above was further centrifuged at 14,000 × *g* for 30 min and resulting pellets were discarded. Next, 0.5 volumes of the total exosome isolation reagent (Life Technologies) were added to the supernatants and incubated at 4 °C overnight. The supernatant from the final step was then centrifuged at 14,000 × *g* for 1 h. In parallel, the supernatant (residuum collected after exosome isolation) was collected. The resulting pellet was resuspended in 25 μL PBS and stored at −80 °C until further analysis.

### Transmission electron microscopy

For electron microscopy, purified EVs were added to 200 mesh formvar-coated grids (Agar Scientific, Essex, UK) and allowed to dry at room temperature. The grids were washed with water and stained with 1% uranyl acetate (System Biosciences, Mountain View, CA, USA) for 5 min. After staining, the grids were washed once in 70% ethanol followed by four washes with molecular grade water. The grids were then loaded onto the sample holder of the transmission electron microscope (Hitachi H-7600, Tokyo, Japan) and exposed to an 80 kV electron beam for image capture.

### Analysis of exosome-like vesicles by SDS-PAGE

The concentrations of purified exosomes were determined using Bradford protein assays (Sangon Biotech, Shanghai, China). Proteins (5 μg) from isolated *S. japonicum* EVs were separated using precast 4–20% polyacrylamide linear gradient gels (Bio-Rad, Hercules, CA, USA). A pre-stained protein standard (Thermo Scientific, Waltham, MA, USA) was used to track protein migration. After running, gels were stained by silver as previously described^46^ and scanned using a Bio-Rad Molecular Imager FX system (Bio-Rad).

### Enzymatic digestion of protein

The purified exosomal samples that dissolved in PBS were diluted in 30 μL SDT buffer (4% SDS, 100 mM DTT, 150 mM Tris-HCl pH 8.0) and boiled for 5 min. The detergent, DTT and other low-molecular-weight components were removed using 200 μl UA buffer (8 M Urea, 150 mM Tris-HCl pH 8.0) by repeated ultrafiltration (Millipore, 10 kD). Subsequently, the samples were carboxymethylated with iodoacetamide (IAA, 50 mM IAA in UA) in darkness for 30 min at room temperature. After 10 min centrifugation at 14,000×g, filters were washed three times with 100 μL UA buffer and then 100 μL of dissolution buffer (50 mM triethylammonium bicarbonate, pH 8.5) twice. The tryptic peptides resulting from the digestion were extracted with 0.1% formic acid in 60% acetonitrile. The extracts were pooled and completely dried using a vacuum centrifuge.

In addition, nine of the gel blocks were excised and each slice was divided into 3 mm sections. Gel slices were destained using 30 mM potassium ferricyanide/100 mM sodium thiosulfate (1:1 v/v) and washed with Milli-Q water. The spots were incubated in 0.2 M NH_4_HCO_3_ for 20 min and then lyophilized. The in-gel proteins were reduced with DTT (10 mM DTT/ 100 mM NH_4_HCO_3_) for 30 min at 56 °C, then alkylated with IAA (50 mM IAA/100 Mm NH_4_HCO_3_) in the dark at room temperature for 30 min. Gel pieces were briefly rinsed with 100 mM NH_4_HCO_3_ and ACN, respectively. Finally, the protein suspension was digested with 2 μg trypsin (Promega, Madison, USA) in 40 μL 25 mM NH_4_HCO_3_ overnight at 37 °C. Peptides were extracted as described above.

### Mass spectrometry

For total exosomal protein identification, liquid chromatography/mass spectroscopy (LC-MS/MS, Thermo Scientific) analysis was performed using a Q Exactive mass spectrometer coupled to an Easy nLC system (Thermo Scientific). Trypsin-digested peptides (~5 μg) were trapped and desalted on Zorbax 300SB-C18 peptide traps (Agilent Technologies, Wilmington, DE) and separated on a C18-reversed phase column (0.15 mm × 150 mm, Column Technology Inc. Fremont, CA). The Easy nLC system (Thermo Scientific) was used to deliver mobile phases A (0.1% formic acid in HPLC-grade water) and B (0.1% Formic acid in 84% acetonitrile) with a linear gradient of 4–50% B (50 min), 50–100% B (4 min), and then 100% B (6 min) at a flow rate of 250 nL/min. To acquire the MS data, a data-dependent top ten method was used, in which the ten most abundant precursor ions were selected for HCD fragmentation. For survey scans (*m/z* 300–1800), the target value was determined based on predictive Automatic Gain Control at a resolution of 70,000 at *m/z* 200 and dynamic exclusion duration of 25 s. Resolution for HCD spectra was set to 17,500 at *m/z* 200. Normalized collision energy was 27 eV and the under fill ratio, which specifies the minimum percentage of the target value likely to be reached at maximum fill time, was defined as 0.1%.

For in gel protein identification, the Ettan^TM^ MDLC controlled by UNICORN^TM^ software (GE Healthcare), a system for automated multi-dimensional chromatography was used for desalting and separation of peptides prior to on-line LTQ Velos (Thermo Scientific) analyses. In this system, peptide mixtures were desalted on RP trap columns (Zorbax 300 SB C18, Agilent Technologies), and then separated on a C18-reversed phase column (0.15 mm × 150 mm, Column Technology Inc. Fremont, CA). Mobile phase A (0.1% formic acid in HPLC-grade water) and mobile phase B (0.1% formic acid in 84% acetonitrile) were selected. Tryptic peptide mixtures were loaded onto the columns, and separation was done at a flow rate of 2 μL/min by using the linear gradient buffer B described above. LTQ Velos (Thermo Scientific) equipped with a micro-spray interface was connected to the LC setup for eluted peptides detection. Data-dependent MS/MS spectra were obtained simultaneously. Each scan cycle consisted of one full scan mass spectrum (*m/z* 300–1800) followed by 20 MS/MS events of the most intense ions with the following dynamic exclusion settings: repeat count 2, repeat duration 30 s, exclusion duration 90 s.

### Data analysis

For total exosomal protein identification, data interpretation and protein identification were performed with the MS/MS spectra data sets using the Mascot search algorithm (v 2.2, Matrix Science) against the UniProtKB Schistosoma database (download at May 11, 2015, with 16,273 entries). The search parameters were trypsin enzyme, two missed cleavages, fixed modifications of carbamidomethyl, variable modifications of oxidation, a fragment ion mass tolerance of 0.10 Da, and peptide tolerance of 20 ppm. Only proteins with at least two peptide (filter by ion score ≥20 and false discovery rate <0.01) uniquely assigned to the respective sequence were considered identified.

For in gel protein identification, MS/MS spectra were automatically searched against the *Schistosoma* database (described above) using the BioworksBrowser rev. 3.3 (Thermo Electron, San Jose, CA, USA). Protein identification results were extracted from SEQUEST out files with BuildSummary. Peptides were constrained to be tryptic and up to two missed cleavages were allowed. Carbamidomethylation of cysteines was treated as a fixed modification, whereas oxidation of methionine residues was considered as variable modifications. The mass tolerance allowed for the precursor ions was 2.0 Da and fragment ions was 0.8 Da. The protein identification criteria were based on Delta CN (≥0.1) and cross-correlation scores (Xcorr, one charge ≥1.9, two charges ≥2.2, three charges ≥3.75. Annotated spectra of proteins and peptides identified are available at ProteomeXchange Consortium (http://www.ebi.ac.uk/pride) with identifier PXD003364 and PXD003368.

### Gene ontology analysis

To obtain the molecular function, protein class, biological process, and pathway of the proteins involved, we searched the consortium databases of the protein classification software PANTHER (http://www.pantherdb.org/) using *Mus musculus* proteins as a reference.

### Small RNA library preparation and bioinformatics analysis

For the analysis of small RNAs, total RNA was extracted from *S. japonicum* secreted EVs using Trizol LS reagent (Life Technologies), and RNA quality was analyzed using an Agilent 2100 system (Agilent Technologies). Next, RNA was size-selected using 15% denaturing PAGE and libraries were prepared from the 18–30 nt fraction and ligated first to a 5′ RNA adaptor and then to a 3′ RNA adaptor using an Illumina small RNA Preparation Kit (version 1, Illumina). The small RNA libraries were subjected to sequencing using Illumina 50 bp single end sequencing performed on an Illumina Hiseq 2000 machine at the BGI (Beijing Genomics Institute, Shenzhen, China). The raw sequencing data were deposited with the NCBI SRA under project number PRJN305851.

After removing of 3′ adaptor null reads, insert null reads, 5′ adaptor contaminants, and reads with a polyA tail, clean datasets were mapped to the draft *S. japonicum* genome sequences (sjr2_scaffold.fasta, downloaded from (ftp://lifecenter.sgst.cn:2121/nucleotide/corenucleotide) using the Short Oligonucleotide Alignment Program (http://soap.genomics.org.cn). In addition, clean datasets were also analyzed by mapping to the rabbit genome (http://asia.ensembl.org/Oryctolagus_cuniculus/, version 81) to identify host information in the *S. japonicum*-secreted EV libraries.

By comparing our sequences with those in databases and selecting the genome location overlap between our data and the databases, sequenced small RNAs were annotated to different categories, including rRNA, tRNA, small nuclear RNA (snRNA), miRNAs, and small nucleolar RNA (snoRNA), by matching against sequences of noncoding RNAs collected in Rfam (Version 11.0) and the NCBI GenBank database (http://www.ncbi.nlm.nih.gov/) using BLAST. For miRNA analysis, these unmatched small RNAs were further analyzed against miRbase (version 21) and GenBank to determine known miRNAs or homologous miRNAs. Finally, unannotated small RNAs were subject to analysis for novel miRNA identification using the Mireap (http://sourceforge.net/projects/mireap). RNAfold was used to predict hairpin-like structures.

### qRT-PCR analysis of *S. japonicum* EV associated miRNAs

A stem-loop based qRT-PCR was used to validate the presence of miRNAs in *S. japonicum* EVs. Small RNAs isolated from *S. japonicum* EVs, culture medium, and residuum supernatants (collected after EVs isolation) were extracted using a mirVana PARIS Kit (Life Technologies) according to the manufacturer’s instructions. In parallel, Sja-miR-1175, which was not found in the *S. japonicum* EV library was used as a negative control. The qRT-PCR analysis was performed as previously described^24^. Briefly, a stem-loop RT primer was used to reverse-transcribe mature miRNAs to cDNAs: (ocu-miR-191, GTC GTA TCC AGT GCA GGG TCC GAG GTA TTC GCA CTG GAT ACG ACC AGC TG; Sja-miR-1175, GTC GTA TCC AGT GCA GGG TCC GAG GTA TTC GCA CTG GAT ACG ACC AGT TG) by using a PrimeScript^TM^ RT Reagent Kit (Takara) according to the manufacturer’s instructions. PCR was performed in a Mastercycler ep realplex (Eppendorf). The 20 μL PCR reaction included 2 μL of RT product (1:3 dilution), 1 × SYBR Premix Ex Taq II (Takara), 0.5 μM specific forward primer (ocu-miR-191_F, ATC GTA CGT GGG CAA CGG AAT C; miR-1175_F, ATC GTA CGT GGG TGA GAT TCA), 0.5 μM common reverse primer (GCA GGG TCC GAG GTA TTC). The abundance of nicotinamide adenine dinucleotide dehydrogenase (*NADH*) (forward primer: CGA GGA CCT AAC AGC AGA GG; reverse primer: TCC GAA CGA ACT TTG AAT CC) was used as the internal control for normalization. The 2^−ΔCt^ method was used to calculate relative miRNA abundance. All reactions were performed in triplicate.

### Analysis of antibody responses to *S. japonicum* EVs

Antibody responses to *S. japonicum* secreted EVs in sera of rabbits (healthy and *S. japonicum*-infected) were evaluated using indirect ELISA and Western blot. Sera from rabbits infected with *S. japonicum* at 45 dpi were collected and pooled (n = 10). An ELISA method was performed as previously described^47^. The *S. japonicum* secreted EVs were coated as antigens on the ELISA plate with 1 μg (100 μL) per well of coating buffer (0.05 M sodium carbonate solution, pH 9.6) and kept at 4 °C overnight. To start the assay, plates were blocked with 5% bovine serum albumin in PBS for 1 h at 25 °C. After the plates were washed three times with PBST, diluted rabbit sera at different dilutions (1:10 and 1:50) in PBS containing 1% bovine serum albumin were added, incubated at 37 °C for 1 h, and washed with 1% Tween in PBS. An HRP-labeled goat anti-rabbit IgG secondary antibody (Kexin Bioscience, China) diluted 1:10,000 was added and incubated at 37 °C for 1 h. Next, the color reaction was developed by adding 3,3′,5,5′-tetramethylbenzidine (TMB) substrate (BiYunTian Biotechnology Research Institute, Nantong, Jiangsu, China) to each well; 50 μL/well of 2 M H_2_SO_4_ was used to stop the reaction. Finally, the optical density (OD) in each well was measured at 450 nm using a Tecan Infinite M200 Pro plate reader (Tecan Group Ltd., Männedorf, Switzerland). The OD 450 values of sample wells above 2.1 times that of the negative control wells were considered positive^47^.

To obtain enough protein for Western blot (30 μg), EVs were pooled from individual harvests (n = 4). The culture media and EV pellets containing 30 μg of protein were lysed with Laemmli sample buffer and run on 10% gels. *S. japonicum* and 293 T cell lysates were used as controls. Proteins were transferred onto a PVDF membrane (Whatman International Ltd., Kent, UK). Membranes were blocked with 5% (w/v) skim milk powder in PBST and probed with primary rabbit serum (1:100, pooled from at least five rabbits at 45 dpi) overnight at 4 °C in 1% milk in PBST, followed by incubation with HRP-conjugated goat anti-rabbit-IgG secondary antibodies (Kexin Bioscience, Shenzhen, Guangzhou, China) at a 1:2000 dilution in 1% milk in PBST for 1 h at room temperature. After extensive washing, the blots were incubated for 1 min at room temperature with Immobilon Western Chemiluminescent HRP Substrate (Millipore) and visualized using Image Quant Las4000 Mini (GE Healthcare Limited).

### Uptake of *S. japonicum* EVs by murine liver cells

Murine liver cells (NCTC clone 1469 cells) were obtained from the ATCC and grown according to the standard protocol using DMEM (Life Technologies) medium supplemented with 10% horse serum (Life Technologies). EVs isolated from the supernatant of NCTC clone 1469 cells were set as a positive control. Briefly, cell supernatants were harvested, centrifuged at 2,000 × *g* for 30 min to eliminate cells, and then centrifuged at 12,000 × *g* for 20 min to eliminate cellular debris and larger vesicles. Exosomes were pelleted using the kit and incubated at 4 °C overnight; then, they were centrifuged at 12,000 × *g* for 60 min at 4 °C. The protein concentration of EVs was determined using a BCA protein assay kit (Sangon Biotech, Shanghai, China).

NCTC clone 1469 cells were seeded in 6-well plates (2 × 10^5^ cells per well) and cultured with advanced DMEM serum-free media (Life Technologies) for 4 h. The isolated exosomes from *S. japonicum* or mouse liver cells (NCTC clone 1469 cells) were labeled with the green fluorescent dye PKH67 (Sigma) as described by Lasser *et al*.^48^ with minor modification. Briefly, 10 μg of the PKH67-stained EVs (extracted from *S. japonicum* culture medium and NCTC clone 1469 cell medium) was washed five times using 300 kDa Vivaspin filters (Sartorius Stedim Biotech GmbH, Goettingen, Germany) to remove excess dye; EVs were then added to the cells and incubated for 1 h at 37 °C. As a control for non-specific labeling of cells, PBS was PKH67-stained, washed, and added to the cells in a parallel experiment. After incubation, medium was aspirated, cells were washed twice with PBS, fixed with 4% formaldehyde solution for 15 min and washed twice more with PBS; nuclei were stained with 4′,6-diamidino-2-phenylindole (DAPI) (Life Technologies). Finally, the cells were observed using fluorescence microscopy (Olympus, Tokyo, Japan).

### qRT-PCR analysis of *Schistosoma*-specific miRNAs

Total RNAs from *S. japonicum* EVs were extracted using TRIzol LS reagent (Life Technologies) as described above. NCTC clone 1469 cells were incubated with *S. japonicum* EVs (10 μg total protein per well) for 20 h. In parallel, exosomes isolated from NCTC clone 1469 cells (10 μg total protein per well) were also incubated with the mouse liver cells as negative control. After washing twice with PBS, total RNAs was isolated from the incubated cells using TRIzol LS reagent. RNA quantity was assessed using a NanoDrop 1000 (Thermo Scientific). A miScript II RT Kit (Qiagen, Hilden, Germany) was used to reverse transcribe RNA to cDNA. Reverse transcription reactions (20 μL) contained 2 μL RNA solution (approximate 60 ng of total RNA), 4 μL 5 × miScript HiFlex buffer, 2 μL miScript Reverse Transcriptase Mix, 2 μL 10 × miScript Nucleics Mix, and 10 μL dH_2_O. Real-time PCR was performed using a miScript SYBR Green PCR Kit (Qiagen) in an Eppendorf Realplex 2 Detection System Mastercycler ep realplex (Eppendorf, Hamburg, Germany)^24^. The PCR reactions were performed at 95 °C for 15 min, followed by 45 cycles of 94 °C for 15 s, 55 °C for 30 s, and 70 °C for 30 s. The miScript primers for Bantam and miR-10 were the property of Qiagen. Glyceraldehyde 3-phosphate dehydrogenase (*GAPDH*) was used as the internal control (forward primer: CAT GGC CTT CCG TGT TCC TA; reverse primer: CCT GCT TCA CCA CCT TCT TGA T). The 2^−ΔCt^ method was used to calculate relative miRNA abundance.

### Bioinformatics analysis of miRNA targets

To determine the potential function of *Schistosoma*-specific miRNAs in host cells, we used miRanda, TargetScan, and RNAhybrid to predict the mouse target mRNAs of Bantam. Mouse mRNA sequences were downloaded from the NCBI databases (ftp://ftp.ncbi.nlm.nih.gov/genomes/M_musculus/RNA/). The predicted target genes were evaluated with the principle by homology, free energy and seed region as previously described^49^. Three genes GINS complex subunit 4 (Sld5 homolog) (*Gins4*), trypsin domain containing 1 (*Tysnd1*), and small subunit (SSU) processome component, homolog (*S. cerevisiae*) (*Utp3*) were selected for further analysis.

### qRT-PCR analysis of the expression of mouse cDNAs that are potentially regulated by *S. japonicum* EV associated miRNAs

Total RNAs from livers of *S. japonicum* infected mice (25 dpi and 35 dpi, pool of five mice, respectively) and uninfected mice were extracted by using TRIzol (Invitrogen), and transcribed into cDNA using a PrimeScript RT reagent Kit (Takara, Japan) and qRT-PCR analysis was performed using the SYBR Premix ExTaq kit (TaKaRa, Japan) according to the manufacturer’s instructions. All PCR reactions were performed in a 20 μL reaction mixture under the conditions: 95 °C for 30 s, 35 cycles at 95 °C for 5 s, 60 °C for 30 s, and 72 °C for 8 s. Primer sequences were as follows: *Gins4*_F, CAG TTC CCA AAC CAG ACC; *Gins4*_R, GTG AGC CCA CCT CCA AGT; *Tysnd1*_F, GTG GGC TTT GGT GTC TTT; *Tysnd1*_R, GCT CCC GTG TTG TTG TCT; *Utp3*_F, GAT TTG GCT AAA GTC TCG; *Utp3*_R, TAC TTA GTC TTC AGG TAT TCG. The *GAPDH* gene was used as the internal control. The relative expression of each gene was calculated as described above.

### Statistical analysis

Results were analyzed using SPSS software (version 17). Comparisons between groups were made using Student’s t-tests. Differences among three groups were evaluated using one-way ANOVA followed by a Tukey’s test. Statistical differences were considered significant when *P* < 0.05. Data are expressed as mean ± SEM.

## Additional Information

**How to cite this article**: Zhu, L. *et al*. Molecular characterization of *S. japonicum* exosome-like vesicles reveals their regulatory roles in parasite-host interactions. *Sci. Rep*. **6**, 25885; doi: 10.1038/srep25885 (2016).

## Supplementary Material

Supplementary Information

Supplementary Dataset S1

Supplementary Dataset S2

Supplementary Dataset S3

Supplementary Dataset S4

## Figures and Tables

**Table 1:** *S. japonicum* EV proteins are homologous to exosomal proteins in other species.

Description of the identified proteins in *S. japonicum* EVs	Homologs of exosomal proteins in other species			E value$	Identities (%)
	Plasma	Cells	Parasites		
Actin	√^50^	√^51^	√^27^	0	91
26 S proteasome regulatory subunit T3		√^52^		0	89
Proteasome (Prosome, macropain) 26 S subunit, ATPase 2			√^27^	0	88
Proteasome (Prosome, macropain) 26 S subunit, ATPase 3		√^52^		0	83
Eukaryotic translation elongation factor 1 alpha 2		√^51^	√^27^	0	81
CDC42 protein	√^50^,^53^			2.00E-109	78
26 S proteasome regulatory subunit N11		√^54^		3.00E-179	78
Glyceraldehyde 3-phosphate dehydrogenase	√^50^	√^51^	√^19^	0	75
Heat shock protein 70	√^53^	√^51^	√^14^,^26^	0	72
Glucose-6-phosphate isomerase	√^53^			0	70
Proteasome subunit beta type 5		√^52^	√^26^	7.00E-102	70
Heat shock protein 90	√^53^	√^52^	√^14^	0	68
Phosphoglycerate kinase		√^54^		0	68
Rab-protein 11		√^54^	√^14^,^19^	2.00E-89	68
Proteasome (Prosome, macropain) subunit alpha type 4		√^51^	√^27^	E-118	67
Glutamine synthetase	√^53^			0	66
26 S proteasome non-ATPase regulatory subunit 7				6.00E-126	66
Rab-protein 10	√^53^	√^54^	√^19^	E-89	65
Tyrosine 3-monooxygenase/tryptophan 5-monooxygenase activation protein, beta polypeptide		√^51^,^54^		6.00E-112	64
Lactate dehydrogenase		√^54^		2.00E-144	61
Proteasome subunit beta type 6			√^26^	2.00E-87	61
20 S proteasome subunit alpha 7		√^52^	√^27^	3.00E-105	60
Proteasome subunit beta type 7		√^54^	√^26^	9.00E-117	60

^$^The protein sequences in *S. japonicum* were analyzed with the homologs in *Mus musculus* by BLASTP program. Blast E value and identities was calculated.

**Table 2:** List of identified miRNAs associated with *S. japonicum* EVs.

Small RNA IDs	Genomic locations				Sequences	Reads*	miRNAs
t0000001	SJC_S000052	314799	314820	+	AACCCTGTAGACCCGAGTTTGG	347581	sja-miR-10-5p
t0000002	SJC_S000383	359175	359196	−	TCCCTGAGACTGATAATTGCTC	260493	sja-miR-125b
t0000121	SJC_S000664	24730	24752	+	TGACTAGAAAGTGCACTCACTTC	9634	sja-miR-61
t0000305	SJC_S000054	245393	245413	−	CGTCTCAAAGGACTGTGAGCC	4215	sja-miR-2b-5p
t0000329	SJC_S000254	288019	288040	+	TGAGATCGCGATTAAAGCTGGT	3990	sja-bantam
t0000380	SJC_S000471	22294	22314	−	TATTATGCAACGTTTCACTCT	3513	sja-miR-2162-3p
t0000902	SJC_S005824	19633	19653	+	GGAGGTAGTTCGTTGTGTGGT	1845	sja-let-7
t0000918	SJC_S004051	17258	17277	+	GGAGGATCGATGAACGGAGC	1837	sja-miR-8185
t0001442	SJC_S000001	925810	925830	−	TAAATGCATTTTCTGGCCCGT	1627	sja-miR-277
t0001782	SJC_S000027	600247	600269	−	CCACCGGGTAGACATTCATTCGC	1489	sja-miR-36-3p
t0001800	SJC_S000136	315901	315920	+	GCCACAACAGTTCGAGGACG	1485	sja-miR-3489
t0001943	SJC_S000102	364554	364577	+	TATCACAGTCCTGCTTAGGTGACG	1389	sja-miR-2d-3p
t0002884	SJC_S001823	36300	36318	+	TCCTCGAACTGTTGTGGCC	910	sja-miR-3487
t0003609	SJC_S000102	364617	364637	+	ACCCTTGTTCGACTGTGATGT	873	sja-miR-2c-5p
t0005423	SJC_S000054	245575	245597	−	TGAAAGACGATGGTAGTGAGATG	804	sja-miR-71a
t0000521	SJC_S000701	78619	78639	+	ACAAGCGTGACTGTCTGGACT	2829	novel-miR-2
t0000605	SJC_S000769	22845	22864	+	TTGCTGGAGGAACCTTGGAC	2577	novel-miR-6
t0001185	SJC_S002785	117	136	+	TACTCTACTTGTCTCTGCCT	1711	novel-miR-8
t0002232	SJC_S001298	37776	37796	+	AGTTCAATCGTGTCGGGTGTT	988	novel-miR-22
t0002527	SJC_S005847	716	735	−	CTTCGGTCGTGTTTCGCTGC	939	novel-miR-26
t0002974	SJC_S002475	9923	9943	+	TAGCTGTAGTTGCAGGTCCCT	905	novel-miR-15
t0003581	SJC_S000112	267998	268018	+	ACGAACCGGACTGAGTTCGAT	874	novel-miR-23
t0003576	SJC_S001710	68535	68556	+	GACACGGAGCGGACTGAGTTCG	874	novel-miR-24
t0003932	SJC_S000418	57959	57979	+	CTGAGCCCCATGATGTCGGTT	860	novel-miR-30
t0004374	SJC_S000483	41623	41642	+	CGGCTGGATGTACCTGCACC	844	novel-miR-12
t0004493	SJC_S000283	212884	212903	+	AGCGAAGACATCTGTAACCA	839	novel-miR-19
t0004827	SJC_S007757	7610	7629	+	CTATCCTGAAGACCTTGGCT	827	novel-miR-5
t0005267	SJC_S000418	62471	62491	−	CTGTCGGTCAAAAAGTGCTCT	810	novel-miR-16
t0005533	SJC_S002283	2741	2760	+	GTCGTAAGCCAGAGTAGGAT	800	novel-miR-18
t0005557	SJC_S000059	843485	843505	+	ACCACGGCCTACTGTAGGACT	799	novel-miR-3
t0007711	SJC_S000913	44846	44865	+	TCGGCTTGTGTTCTTATTCC	671	novel-miR-10
t0007806	SJC_S001339	60022	60044	+	GAACCGGACTGAGTTCGAATCCT	660	novel-miR-20
t0008372	SJC_S015651	593	612	+	TACCGTTCGAACTAATCTAC	557	novel-miR-25
t0008734	SJC_S019902	653	675	+	GAGATGATGATTGGAGTGATTGT	416	novel-miR-9
T000027	9	16649791	16649813	−	CAACGGAATCCCAAAAGCAGCTG	4548	Ocu-miR-191-5p

^*^Only miRNAs with reads >100 were listed out.
