# Supplementary material for: Molecular characterization of S. japonicum exosome-like vesicles reveals their regulatory roles in parasite-host interactions
Source: Sci Rep. 2016 May 13;6:25885. doi: 10.1038/srep25885 (PMC4865838; doi:10.1038/srep25885)
Supplement: Supplementary Information [file srep25885-s1.pdf]

Supplementary information

Molecular characterization of *S. japonicum* exosome-like vesicles reveals their regulatory roles in parasite-host interactions

Lihui Zhu[1], Juntao Liu[1], Jinwei Dao[1], Ke Lu[1], Hao Li[1], Huiming Gu[1], Jinming Liu[1], Xingang Feng[1], & Guofeng Cheng[1]*

[1]Shanghai Veterinary Research Institute, Chinese Academy of Agricultural Sciences, Key Laboratory of Animal Parasitology, Ministry of Agriculture, 518 Ziyue Road, Shanghai, China.

*To whom correspondence may be addressed:

Shanghai Veterinary Research Institute, Chinese Academy of Agricultural Sciences; Key Laboratory of Animal Parasitology, Ministry of Agriculture, 518 Ziyue Road, Shanghai, 200241, China.

Tel: 86-213-429-3659; Fax: 86-215-408-1818;

E-mail: cheng_guofeng@yahoo.com or chenggfeng@shvri.ac.cn.

Supplementary Table S1: The list of structural-associated proteins in *S. japonicum* EVs putatively identified by bioinformatics

Proteins	Database ID no.
Microtubule-associated protein futsch	C1L4F7
Microtubule-associated protein RP/EB family member 1	C1L436
Tubulin, alpha 1	C1LE18
Tubulin beta-2C chain	C1LVE4
Elongation factor 1-alpha	Q5DGS7
Elongation FacTor	C1LFJ1
Eukaryotic translation elongation factor 1 alpha 2	C1LBW3
Eukaryotic translation elongation factor 1 gamma	C1LV11
Eukaryotic translation elongation factor 1 beta 2	C1L985
Putative ribosomal protein L5	C1LUC1
40S ribosomal protein SA	RSSA
40S ribosomal protein S12	Q5DE28
Gelsolin	C1L4M3
Dynein light chain 1	Q9XTK6
Actin-related protein 2/3 complex subunit 3	C1L5L1
Putative Ezrin	C7TXW6
Dynamin 1-like protein	C1LEY9
Ubiquitin carboxyl-terminal hydrolase	C1LIT8
Ubiquitin fusion degradation protein 1 homolog	C1LJP2
Ubiquitin C	C1LYC1
Actin related protein 2/3 complex, subunit 2	C1L4E5
Actin related protein 2/3 complex, subunit 4	Q5DFU4
CDC37 cell division cycle 37 homolog	Q5DGJ2
Laminin, beta 1	C1LED3
Sorting nexin-6	C1LD78
Vacuolar protein sorting-associated protein 28 homolog	Q5DGV7
Sorting nexin-18	C1LG69

Supplementary Table S2: The list of pathways analyses by KEGG

Pathways	Hits	Percent (%)	Genes
Integrin signalling pathway	8	4.20%	ARPC2 TLN1 Pxn ARPC3 SNX6 Lamb1 CDC42 Pik3r1
Ubiquitin proteasome pathway	8	4.20%	PSMD12 PSMC4 PSMD4 PSMD6 PSMD7 PSMC3 PSMC2 PSMD2
CCKR signaling map	8	4.20%	Pxn MAP2K6 PRKG1 YWHAB CDC42 Pik3r1 PTPN11 ROCK1
Huntington disease	7	3.70%	DYNLL1 ARL4A GAPDH DYNC1I2 TUBB4B CAPN10 RpL12
Cytoskeletal regulation by Rho GTPase	7	3.70%	ARPC2 ARPC3 ARPC4 TUBB4B CDC42 ROCK1 RpL12
Gonadotropin releasing hormone receptor pathway	7	3.70%	Pxn MAP2K6 CDC42 Pik3r1 PRKAG2 ANXA5 TUBA1B
Inflammation mediated by chemokine and cytokine signaling pathway	5	2.60%	ARPC2 ARPC3 ARPC4 CDC42 ROCK1
Parkinson disease	5	2.60%	CUL1 YWHAB PSMB4 CSNK2A1 TH
Glycolysis	4	2.10%	GAPDH ALDOA ENO1 GPI
Angiogenesis	3	1.60%	Pxn Pik3r1 PTPN11
Adenine and hypoxanthine salvage pathway	3	1.60%	HPRT1 PNP ADA
FGF signaling pathway	3	1.60%	MAP2K6 YWHAB PTPN11
Endothelin signaling pathway	3	1.60%	PRKAR1A PRKG1 Pik3r1
Cell cycle	3	1.60%	PSMD12 PSMD4 PSMD7
Axon guidance mediated by netrin	2	1.00%	CDC42 Pik3r1
Pentose phosphate pathway	2	1.00%	GPI TALDO1
VEGF signaling pathway	2	1.00%	Pik3r1 Pxn
Ras Pathway	2	1.00%	MAP2K6 CDC42
T cell activation	2	1.00%	CDC42 Pik3r1
EGF receptor signaling pathway	2	1.00%	MAP2K6 YWHAB
p38 MAPK pathway	2	1.00%	MAP2K6 CDC42
PDGF signaling pathway	2	1.00%	ARHGAP12 Pik3r1
Oxidative stress response	2	1.00%	TXN MAP2K6

Supplementary Table S3: The list of membrane- and tegument-associated proteins in *S. japonicum* EVs putatively identified by bioinformatics

Proteins	Database ID no.	Molecular functions	Biological processes
6-phosphofructokinase	C1LED7		
22.6 kDa tegumental antigen	O44372		
Actin 5C	Q5DF69		
Actin related protein 2/3 complex, subunit 2	C1L4E5	Structural molecule activity	
Actin related protein 2/3 complex, subunit 4	Q5DFU4	Structural molecule activity	
Actin-like protein 3	C1LJL2	Structural molecule activity	
Actin-related protein 2/3 complex subunit 3	C1L5L1	Structural molecule activity	
Alpha-centractin (Centractin) (Centrosome-associated actin homolog) (Actin-RPV) (ARP1) (Fragment)	C7TQQ1		
Ago2	M4QLF4	Catalytic activity	
Calcium-binding EF-hand,domain-containing protein	Q5BQX8	Calcium binding	Calcium ion transport
Calcium-binding EF-hand,domain-containing protein	C1LEK4	Calcium binding	Calcium ion transport
CD63 antigen	F6LHR8	Calcium binding	Calcium ion transport
Dynactin subunit 2	C1LHQ2		
Dynactin subunit 4	C1LGU5		
Dynein light chain	C1L712		
Dynein light chain	Q9XTL4		
Dynein light chain	Q86H14		
Dynein light chain 1	Q9XTK6	Enzyme regulator activity	
Elongation FacTor	C1LFJ1	Structural molecule activity	
Elongation factor 1-alpha	Q5DGS7	Nucleotide-binding	Protein biosynthesis
Eukaryotic translation elongation factor 1 alpha 2	C1LBW3	Translation regulator activity	
Eukaryotic translation elongation factor 1 beta 2	C1L985	Translation regulator activity	
Eukaryotic translation elongation factor 1 gamma	C1LV11	Translation regulator activity	
Fatty acid binding protein 7, brain	C1L9V6	Lipid binding	
Fructose-bisphosphate aldolase	Q1HDV2		
Glutathione S-Transferase	Q26513		Detoxification
Glyceraldehyde-3-phosphate dehydrogenase	C7TRL1	Catalytic activity	Glycolysis
Heat shock protein 60 (Fragment)	C7TZI6		Stress response
Major egg antigen (P40)	C1LU54		
MF3 protein (Fragment)	Q8T5U1		
Mitochondrial import receptor subunit TOM34	C1LQQ3		
Phosphoglycerate mutase	C1LNQ1		Glycolysis
Phosphorylase	C1L566		Carbohydrate

			metabolism
Putative actin-interacting protein 1	C1LJU4		
Putative dynein light chain	Q5BS76		
Putative dynein light chain	Q5BQQ3		
Pyruvate kinase	Q5DAM7		
Tegument antigen (I(H)A)	C1L468		
Tropomyosin-2	C7TXT8		Muscle contraction
Tubulin beta-2C chain	C1LVE4	Structural molecule activity	Microtubule-based process
Heat shock protein 90	Q5D947		Stress response
Putative ribosomal protein L5	C1LUC1	Structural molecule activity	
40S ribosomal protein SA	Q5DA90	Structural molecule activity	
40S ribosomal protein S12	Q5DE28	Structural molecule activity	
T-complex protein 1 subunit delta	C1LI13		
T-complex protein 1 subunit alpha	Q5DF55		
T-complex protein 1 subunit gamma	C1LHD3		
Ubiquitin C	C1LYC1	Protein binding	Protein modification process
Ubiquitin carboxyl-terminal hydrolase	C1LIT8	Protein binding	Protein modification process
Ubiquitin fusion degradation protein 1 homolog	C1LJP2	Protein binding	Protein modification process

Supplementary Table S4: The List of identified miRNAs associated with *S. japonicum* EVs homologous to miRNAs deposited in ExoCarta

Small RNA IDs	Sequences	miRNAs	miRNAs deposited in Exocarta	E-value$
t0000001	AACCCTGTAGACCCGAGTTTGG	sja-miR-10-5p	hsa-miR-10a-5p	0.13
t0000305	CGTCTCAAAGGACTGTGAGCC	sja-miR-2b-5p	hsa-miR-28-5p	2.2
t0000902	GGAGGTAGTTCGTTGTGTGGT	sja-let-7	hsa-let-7b-5p	0.12
T000027	CAACGGAATCCCAAAAGCAGCTG	Ocu-miR-191-5p	hsa-miR-191-5p	2e-04

$The miRNA sequences in *S japonicum* were analyzed with homologous to *Homo sapiens* miRNAs deposited in Exocarta database by BLASTN program. Blast E value was calculated.
